# Interpersonal psychotherapy for perinatal women: a systematic review and meta-analysis protocol

**DOI:** 10.1186/s13643-019-1158-6

**Published:** 2019-10-29

**Authors:** Katherine S. Bright, Elyse M. Charrois, Muhammad Kashif Mughal, Abdul Wajid, Deborah McNeil, Scott Stuart, K. Alix Hayden, Dawn Kingston

**Affiliations:** 1 Faculty of Nursing, University of Calgary, PF2226, 2500 University Drive NW, Calgary, Alberta T2N 1N4 Canada; 2 Alberta Children’s Hospital Research Institute (ACHRI), Calgary, Alberta Canada; 3 Alberta Health Services, Southport Atrium #2237, 10101 Southport Road, S.W., Calgary, Alberta Canada; 4 Psychiatry/Psychological & Brain Sciences, University of Iowa, 1-293 MEB, W311 Seashore Hall, Iowa City, IA 55241-1407 USA; 5 Libraries and Cultural Resources, University of Calgary, 2500 University Dr. N.W., Calgary, Alberta T2N 1N4 Canada

**Keywords:** Systematic review, Interpersonal psychotherapy, Antenatal, Perinatal, Postpartum, Women, Distress

## Abstract

**Background:**

Interpersonal psychotherapy (IPT) is an intervention that has established efficacy in the prevention and treatment of depressive disorders. Previous systematic reviews have not evaluated the effectiveness of IPT on symptoms of stress, anxiety, depression, quality of life, relationship satisfaction/quality, social supports, and an improved psychological sense of well-being. There is limited data regarding factors that moderate and mediate the effectiveness of IPT including the timing of the intervention or the mode of delivery of IPT intervention. The objective of this systematic review and meta-analysis is to evaluate the effectiveness, feasibility, and acceptability of IPT interventions to treat perinatal psychological distress and to summarize the evidence on predictors, mediators, and moderators of IPT.

**Methods:**

We will include peer-reviewed studies that recruited perinatal women. The search strategy will involve the following databases: MEDLINE (Ovid), EMBASE (Ovid), PsycINFO (Ovid), Cochrane Central Register of Controlled Trials (Ovid), CINAHL with Full Text (EBSCO), Social Work Abstracts (EBSCO), SocINDEX with Full Text (EBSCO), Academic Search Complete (EBSCO), Family & Society Studies Worldwide (EBSCO), Family Studies Abstracts (EBSCO), and Scopus. Study inclusion criteria include (1) randomized controlled trials, quasi-experimental studies, and pre-post studies that evaluated the effectiveness of IPT; (2) qualitative studies that evaluated feasibility and acceptability of IPT; (3) study sample included and analyzed perinatal women; and (4) publication language was English. Using pilot-tested screening and data extraction forms, two reviewers will independently review studies in three steps: (1) abstract/title screening, (2) full-text screening of potentially accepted studies, and (3) data extraction of accepted studies. Disagreements will be resolved by a third reviewer. Studies will be aggregated for meta-synthesis and meta-analysis should the data allow for this. Two independent reviewers will grade methodological quality.

**Discussion:**

Findings from this review will inform future development and implementation of IPT intervention research for perinatal women. Identifying key factors of successful IPT interventions will inform intervention design and adaptation of IPT interventions to increase the likelihood that perinatal women will engage in and benefit from IPT interventions. This review will also identify key considerations for increasing the effectiveness of IPT interventions during the perinatal period.

**Systematic review registration:**

PROSPERO CRD42019114292

## Background

The perinatal period, from conception until 12-months postpartum, is a time of increased social, emotional, biological, and psychological adjustments [1, 2]. Additionally, the perinatal period is a developmental life stage involving significant adaptation to changes in physical appearance and expectations for new roles [3, 4]. The perinatal period is also a time of increased vulnerability to psychological stress and an impaired sense of well-being [5, 6]. Epidemiological research suggests that psychological distress, including stress, anxiety, and depression, resulting from these changes occurs between 15 and 25% of perinatal women [7, 8]. Psychological distress during the perinatal period is also associated with impaired mother-fetal/infant relationship, obstetrical complications, and child developmental problems [9, 10]. Additionally, left untreated, up to 40% of these women will have symptoms that persist until their children enter preschool and beyond [11, 12]. However, psychological distress during the perinatal period is often under detected and untreated [13–15].

The impact of perinatal psychological distress on infants, children, women, and their families is well established. Evidence regarding the most effective psychological treatments during the perinatal period is emerging [16, 17]. There are over 35 years of research examining the use of cognitive behavior therapy (CBT) and interpersonal psychotherapy (IPT) in adult populations with the results suggesting that these treatments are equally efficacious in treating depression in the short term [18, 19] and the long term [20]. Studies examining the efficacy of IPT during the perinatal period are also promising. One pilot RCT compared the efficacy of 16 45-min individualized session in IPT (21 participants) with a parenting education program (17 participants) for new immigrants from the Dominican Republic [21]. The IPT treatment group showed significant improvements in mood compared to the parenting education control program [21]. While attrition rate in the study was high and there was greater homogeneity of the sample population both which significantly limited the generalizability of these findings, the outcome of improvements in mood in the IPT treatment group are promising [21].

An American study of group IPT for depressed pregnant women receiving public assistance found that at 3 months postpartum, no one in the IPT group had symptoms of depression compared to 33% of women in the treatment-as-usual (no therapy) group [22]. In another American study exploring group IPT in pregnant women, IPT was found to be an effective treatment for the reduction of depression symptoms in pregnancy and prevention of postpartum depression [23]. To date, studies examining IPT treatment in pregnancy are limited by their small sample sizes and thus reduced generalizability of their findings.

IPT is considered the best front-line treatment for postpartum depression (PPD) when symptoms are mild and in conjunction with antidepressant medications when symptoms are in the moderate to severe range [24–27]. In a systematic review from 2014, 11 studies summarizing the efficacy of IPT for PPD found support for both individual and group IPT interventions for women [28]. The systematic review suggested that women who engaged in IPT noted significant improvements in their marital and mother-newborn relationships. Other studies report significant improvement in depressive symptoms [29, 30].

Delivered to postpartum women, IPT focuses on increasing social supports while addressing interpersonal problems that result from a lack of help with childcare or a perceived absence of emotional support [24]. The transition into motherhood may involve decisions about new roles and focusing attention in new areas [24]. IPT during the postpartum period also may address components of grief and loss such as miscarriage, loss of independent identity, loss of employment, and closeness in intimate relationship [24].

In a recent systematic review (2018) of the efficacy of IPT in perinatal women, 28 studies endorsed the effectiveness of IPT in the prevention and/or treatment of perinatal distress [31]. The 2018 review lacked congruency with systematic literature review best practices as the search was limited to two databases, screening was completed by only one reviewer, and we were not able to replicate the search strategy results [31]. Thus, there is a need for a more systematic, comprehensive, and transparent approach to examining the use of IPT in perinatal women.

While evidence suggests that psychological therapy is effective, perinatal women report significant barriers in seeking psychological support including stigma (self and by their healthcare professional), uncertainty about whether their symptoms are normal or abnormal, inability to articulate their distress, wanting to self-manage first, not wanting to take psychotropic medications, lack of time, financial expenditure, location and proximity of services, transportation issues, and challenges associated with childcare [8, 14, 32]. As a result, instead of using formal treatments, women are more inclined to seek informal support from family, printed material, or computer/web-based intervention programs [7, 8, 33].

Individual characteristics that influence computer/web-based treatment effectiveness can be categorized into predictors, moderators, and mediators [34]. Predictors and moderators are considered pre-treatment variables; however, the former forecasts mental health outcomes in the treatment groups, while the latter identifies persons more likely to benefit from which particular treatment [34]. Examples of predictors of computer/web-based interventions include when the therapy is guided by a coach/therapist, being female, and obtaining low mastery scores and dysfunctional attitudes [34, 35]. Interestingly, a moderator of internet-based therapy is age, with older adults benefiting more from CBT and younger adults noting more significant improvements with IPT [34]. For younger adults, the improvements from internet-based IPT are due to the foci of IPT; specifically, interpersonal conflicts and role transitions are particularly relevant to the stage of life for young adults [24, 34].

Additionally, IPT is an intervention aimed at alleviating psychological symptoms, coping with problems due to loss, change, and relationship conflict, thereby improving interpersonal functioning [36, 37]. IPT is a therapy based on the notion that when faced with adversity, factors such as attachment needs, communication patterns, and the quality of social support networks contribute significantly to an individual’s range of symptoms of psychological distress [37]. Conceptualizations of social supports come from work on attachment theory, trust, and coping in times of adversity [38]. These social supports play an important role in how individuals manage stress and work through the coping process [39–43]. IPT attempts to improve attachment security, interpersonal change, and psychological distress [44, 45]. As a result, IPT has the potential to improve individual coping and resiliency.

### Objectives

The objective of this review is to evaluate the effectiveness, feasibility, and acceptability of IPT interventions to treat perinatal psychological distress and to summarize the evidence on predictors, mediators, and moderators of IPT. The questions guiding this systematic review and meta-analysis are the following:

1. What is the effectiveness of IPT for women during the perinatal period on the reduction of stress, anxiety, depression, quality of life, relationship satisfaction/quality, social support, and improved psychological well-being?
2. What are the predictors, mediators, and moderators of IPT, including timing of IPT and mode of delivery?
3. What is the feasibility and acceptability of IPT?

## Methods

### Protocol and registration

The protocol for this systematic review was developed according to the Preferred Reporting Items for Systematic reviews and Meta-analyses Protocols (PRISMA-P) [46] (see Additional file 1) and has been registered with PROSPERO CRD42019114292. We will document any amendments to the protocol with a rationale and report them with the final publication.

### Eligibility criteria

The studies selected for inclusion in this systematic review will meet the following eligibility criteria which are described according to participants, study design (including publication, language, and year), intervention, and outcomes.

#### Participants

Perinatal women from conception to 12 months postpartum who participated in IPT intervention. For this review, women who are not pregnant or postpartum or men who engage in IPT interventions will be excluded. The rationale for this exclusion is that we are exploring the effectiveness of IPT for women during the perinatal period.

#### Study design

The review will consider studies evaluating the feasibility, acceptability, effectiveness, and/or efficacy of IPT in perinatal women. Experimental studies such as randomized controlled/clinical trials (RCTs), quasi-experimental studies, as well as single-group pre-post studies will be included in the review. In cases of duplicate publications of the same sample, we will include the publication with the largest sample size. If sample sizes are consistent, the first published study from the sample will be included. We will also include qualitative studies that explore the acceptability of the intervention. We will exclude conference papers, dissertations, reviews, and non-English publications.

#### Interventions

We define IPT intervention as an intervention, which may include interpersonal therapy, or intervention, counseling, psychotherapy, therapy, or program where there was a component of IPT offered. IPT will include those interventions targeted towards women during the perinatal period. Interventions not including IPT will be excluded.

#### Comparator

We will include studies with all types of comparator groups, such as non-exposed control group or a group exposed to a different intervention.

#### Outcomes

##### Primary outcome

Similar to other reviews on the effectiveness of IPT for women during the perinatal period, this review will synthesize information on the effectiveness of IPT for a range of outcomes including stress, anxiety, depression, quality of life, relationship satisfaction/quality, social support, and improved psychological well-being. Effect sizes will be calculated based on within-group effect size and between-group effect size.

##### Secondary outcomes

This review will also synthesize information on predictors, mediators, and moderators of IPT, including timing of IPT and mode of delivery. Predictors of treatment efficacy will include clinical or biological factors such as age and history of mental health concerns, whereas mediators of treatment efficacy measure changes that occur during treatment and these mediators correlate with treatment outcome. Examples of mediators of treatment may include interpersonal conflict and maladaptive communication skills. According to the theoretical framework, IPT works by virtue of improving conflict in relationships by improving communication skills [37]. Moderators of treatment efficacy outcomes also involves clinical or biological factors, these include the presence or magnitude of a factor at baseline that influences the relative likelihood of a particular outcome occurring following treatment with one versus another agent.

##### Tertiary outcomes

Additional outcomes of feasibility and acceptability of IPT will be synthesized. Feasibility will consider whether women find the IPT intervention as easy to complete. Acceptability will be measured by women’s perceptions of the appropriateness of the content; the intervention was found to be effective in achieving the intended outcomes.

### Information sources and search strategy

MEDLINE(R) and Epub Ahead of Print, In-Process & Other Non-Indexed Citations and Daily (Ovid); EMBASE (Ovid); PsycINFO (Ovid); Cochrane Central Register of Controlled Trials (Ovid); CINAHL with Full Text (EBSCO); Social Work Abstracts (EBSCO); SocINDEX with Full Text (EBSCO); Academic Search Complete (EBSCO); Family & Society Studies Worldwide (EBSCO), Family Studies Abstracts (EBSCO); and Scopus databases will be searched from database inception onwards. The search strategy was developed in collaboration with an expert health sciences librarian (KAH). Keywords will be constant across databases, and subject headings will be responsive to the controlled vocabulary of each database. The MEDLINE (Ovid) search strategy is in Table 1. In addition, we will conduct citation searches in Google Scholar, search the reference lists of included studies, and ask experts to identify further studies for inclusion. No search filters or hedges will be used. Article language will be restricted to English. EndNote X8 will be used to manage the records, remove duplicates, and retrieve full-texts. Any full texts missing in Endnote will be retrieved manually. We will re-run the searches prior to the final analyses to retrieve any addition recently published articles for inclusion.

**Table 1:** Search strategy

#	Searches	Results
Database(s): Ovid MEDLINE(R) and Epub Ahead of Print, In-Process & Other Non-Indexed Citations and Daily 1946 to January 28, 2019		
1	exp Prenatal Care/	25,200
2	exp Postnatal Care/	5160
3	exp Postpartum Period/	61,051
4	exp Peripartum Period/	932
5	exp Pregnancy/	852,157
6	(perinatal or prenatal or antenatal or peri-natal or pre-natal or ante-natal or postnatal or post-natal).tw,kf.	255,957
7	(peripartum or peri-partum or intrapartum or intra-partum or antepartum or ante-partum or postpartum or post-partum or puerperium or puerperal).tw,kf.	82,521
8	pregnanc*.tw,kf.	411,047
9	pregnant.tw,kf.	166,554
10	or/1-9	1,104,506
11	((interpersonal or inter-personal) adj2 (therap* or psychotherap* or psycotherap* or psycho-therap* or counsel*)).tw,kf.	1421
12	ipt.tw,kf.	2110
13	ipt-g.tw,kf.	17
14	or/11-13	3068
15	10 and 14	291
Database(s): EMBASE 1974 to 2019 January 30		
1	exp prenatal care/ or exp prenatal period/	148,284
2	exp postnatal care/	102,698
3	exp puerperium/	56,611
4	exp perinatal period/	31,406
5	pregnancy.mp.	798,101
6	exp pregnancy/	622,925
7	(perinatal or prenatal or antenatal or peri-natal or pre-natal or ante-natal or postnatal or post-natal).tw,kw.	324,394
8	(peripartum or peri-partum or intrapartum or intra-partum or antepartum or ante-partum or postpartum or post-partum or puerperium or puerperal).tw,kw.	98,238
9	pregnanc*.tw,kw.	497,235
10	pregnant.tw,kw.	214,578
11	or/1-10	1,138,175
12	((interpersonal or inter-personal) adj2 (therap* or psychotherap* or psycotherap* or psycho-therap* or counsel*)).tw,kw.	1889
13	ipt.tw,kw.	2749
14	ipt-g.tw,kw.	13
15	or/12-14	4066
16	11 and 15	455
Database(s): EBM Reviews - Cochrane Central Register of Controlled Trials December 2018		
1	exp Prenatal Care/	1224
2	exp Postnatal Care/	352
3	exp Postpartum Period/	1388
4	exp Peripartum Period/	7
5	exp Pregnancy/	19,347
6	(perinatal or prenatal or antenatal or peri-natal or pre-natal or ante-natal or postnatal or post-natal).tw,kw.	10,453
7	(peripartum or peri-partum or intrapartum or intra-partum or antepartum or ante-partum or postpartum or post-partum or puerperium or puerperal).tw,kw.	7654
8	pregnanc*.tw,kw.	26,562
9	pregnant.tw,kw.	11,671
10	or/1-9	45,654
11	((interpersonal or inter-personal) adj2 (therap* or psychotherap* or psycotherap* or psycho-therap* or counsel*)).tw,kw.	726
12	ipt.tw,kw.	548
13	ipt-g.tw,kw.	8
14	or/11-13	970
15	10 and 14	115
Database(s): PsycINFO 1806 to January Week 4 2019		
1	exp PRENATAL CARE/	1933
2	exp POSTNATAL PERIOD/	4204
3	(perinatal or prenatal or antenatal or peri-natal or pre-natal or ante-natal or postnatal or post-natal).tw,id.	43,526
4	(peripartum or peri-partum or intrapartum or intra-partum or antepartum or ante-partum or postpartum or post-partum or puerperium or puerperal).tw,id.	12,895
5	pregnanc*.tw,id.	37,518
6	pregnant.tw,id.	17,640
7	or/1-6	80,027
8	exp Interpersonal Psychotherapy/	1283
9	((interpersonal or inter-personal) adj2 (therap* or psychotherap* or psycotherap* or psycho-therap* or counsel*)).tw,id.	3014
10	ipt.tw,id.	1094
11	ipt-g.tw,id.	24
12	or/8-11	3536
13	7 and 12	156

### Screening of studies

Prior to screening titles and abstracts, we will conduct training and an inter-relater calibration exercise with the review team. These two reviewers (KSB and EML), who are experts in perinatal mental health, will then independently screen the remaining studies for eligibility in two steps. The first step will consist of reviewing all studies’ titles/abstracts to identify studies that meet the eligibility criteria. The second step will consist of reviewing the provisionally included studies’ full-text to ensure that they meet all the inclusion criteria. Any disagreements will be resolved by a third expert reviewer (MKM). The total number of studies retrieved, reviewed, included, and excluded as well as reasons for exclusion will be reported.

### Data items and data extraction

Table 2 details the data items to be extracted from the studies. These items are informed by the Template for Intervention Description and Replication (TIDieR) [47] and Transparent Reporting of Evaluations with Nonrandomized Designs (TREND) checklists [48]. Extracted data will include study characteristics, participants, intervention characteristics (including length, timing, mode of delivery, and intensity), participant flow, assignment methods, recruitment methods, and retention methods. We will use a templated Microsoft Excel data extraction tool. After the team completes the inter-rater exercise with the extraction tool, one reviewer (MKM) will extract all study data with a second reviewer verifying the extracted data. Any discrepancies in extracted data will be resolved through discussion.

**Table 2:** Data extraction categories for eligible studies

Category	Data extracted
Study characteristics	First author, year, and country
Participant	Eligibility criteria (including different levels in recruitment/sampling plan), demographics, and data collection setting
Intervention characteristics	Content, delivery method, unit of delivery, deliverer, setting, exposure quantity and duration, and time span
Assignment method	Unit of assignment
	Method used to assign units to study conditions, including details of any restriction
	Inclusion of aspects employed to help minimize potential bias comparison condition induced due to nonrandomization
Participant flow	Flow of participants: enrollment, assignment, allocation and intervention exposure, baseline and outcome measures, and follow-up
Recruitment	Method of recruitment, sampling method, and recruitment setting
Retention	Method to increase compliance or adherence
Outcomes	Stress, anxiety, depression, quality of life, relationship satisfaction/quality, social support, and improved psychological well-being

### Risk of bias in individual studies

Studies will be included regardless of methodological quality. The Cochrane risk of bias tool [49] will be used to rate selection, performance, detection, attrition, reporting, and other biases in each study as either high, unclear, or low risk of bias. For non-randomized studies of interventions, specific to cohort studies, ROBIN-1 will be used to assess bias including overall, selection of participants, type of intervention, deviation from intended intervention, outcome measurements, and reporting of results in studies as either low risk, moderate risk, serious risk, critical risk, or no risk [50]. Two reviewers will independently assess all studies for quality, and disagreements will be resolved through a third expert if a disagreement cannot be solved among the two initial reviewers.

### Data synthesis

Synthesis of the data will be conducted according to the Cochrane guidance [51]. Should the data permit, a meta-analysis will be conducted using Comprehensive Meta-Analysis (CMA) software, version 3.0. Additionally, should data permit, a meta-synthesis will be conducted on the qualitative studies.

Recruitment and retention will be calculated as a proportional effect size with confidence intervals reported based on a 95% criterion. These proportions will be computed first as a proportional effect size and then transformed into logit units using CMA, which allows for more variability in the data than the bound proportional effect sizes [52, 53]. Recruitment rates will be calculated using previously described approaches [54–56] that calculate a rate based on the number of participants who started participation at each recruitment site divided by the total recruitment period (defined as the time in months between the start and completion of recruitment). We will calculate retention based on a previously described strategy [57], in which the number of participants (perinatal women) retained in perinatal IPT research until the time point of primary assessment outcome was collected is compared to the total number of participants enrolled. To calculate attrition rates, we will calculate the number of perinatal women lost during follow-up out of duration between follow-up time points. Feasibility will also consider whether women find the IPT intervention as easy to complete. Acceptability will be measured by women’s perceptions of the appropriateness of the content, found the intervention effective in achieving the intended outcomes.

We will also conduct a sensitivity analysis by sequentially removing one study at a time and reanalyzing the dataset to determine the impact of any given single study. In conjunction with the quality analysis previously mentioned, this procedure allows for the inclusion of methodologically flawed studies if they meet this criterion. Provided that there is enough information, we will also carry out meta-regression using logit units to explore potential modifiers of recruitment and retention rates, such as (a) study characteristics, (b) recruitment methods, (c) retention methods, (d) maternal and family characteristics, and (e) intervention characteristics. In the case that the included studies are too heterogeneous for meta-analysis, we will aggregate findings using narrative synthesis [58].

### Missing data

Authors of studies will be contacted to obtain missing data.

### Assessment of heterogeneity

Heterogeneity of the studies’ recruitment and retention proportions will be assessed by the non-parametric Cochrane *Q* test, which assesses variance between studies and study populations. The *I*^2^ index will be calculated to evaluate the proportion of heterogeneity between studies. If heterogeneity is present, random-effect models (as opposed to fixed-effect models) will be used as they are a more appropriate computational approach under conditions of heterogeneity given they are less likely to reject the null hypothesis. Further, random-effect models are more robust to large variations in sample sizes [59].

### Subgroup analysis

Data permitting, subgroup analysis will be carried out considering participant characteristics (such as stage in pregnancy/postpartum, maternal age, previous children, previous losses) and intervention characteristics (when it was delivered, mode of delivery, dose, frequency, and duration).

### Meta-analysis of bias

Publication bias and selection of variables in publications will be assessed through visual inspection of a funnel plot as well as statistical tests (e.g., Egger’s regression intercept, Begg and Mazumdar’s rank correlation, and Orwin’s fail-safe *N*) [60–62].

## Discussion

Findings from this review will inform future development and implementation of IPT intervention research for perinatal women. Identifying key factors of successful IPT interventions will enable the design and adaptation of IPT interventions to increase the likelihood of perinatal women engaging in and benefiting from IPT interventions during the perinatal period. Researchers will be able to use this review to inform future research that addresses current evidence gaps of IPT interventions for women during pregnancy and 1 year postpartum. This review will also identify key mediators and moderators of IPT interventions to enhance design, implementation, and uptake of IPT.

Limitations to the review may include limited data that could impede the ability to run meta-analyses on all potential sub-groups of participant and study characteristics. Additionally, there may be limited descriptions of recruitment and retention strategies as well as timing of the intervention in the articles under review. Further limitations are related to the inclusion/exclusion criteria of reviewing only English language articles, which may reduce generalizability to non-English-speaking populations. Similarly, the inclusion of only peer-reviewed literature excludes government reports, dissertations, conference papers, and reviews thereby potentially limiting grass-roots or community-based recruitment and retention strategies that may be used to target smaller or marginalized groups of perinatal women.

## Supplementary information

**Additional file 1:** PRISMA-P 2015 Checklist.

## Data Availability

The datasets created and analyzed during this review will be available from the corresponding author upon reasonable request. We will also deposit our datasets in an open access repository, Open Science Framework (https://osf.io/), developed and maintained by the Centre for Open Science.

## Abbreviations

PRISMA-P: Preferred Reporting Items for Systematic reviews and Meta-analyses Protocols
TIDieR: Template for Intervention Description and Replication
TREND: Transparent Reporting of Evaluations with Nonrandomized Designs
